# Characterization and role exploration of ferroptosis-related genes in osteoarthritis

**DOI:** 10.3389/fmolb.2023.1066885

**Published:** 2023-03-06

**Authors:** Xinyu Wang, Tianyi Liu, Cheng Qiu, Shunan Yu, Yanzhuo Zhang, Yueyang Sheng, Chengai Wu

**Affiliations:** ^1^ Department of Molecular Orthopaedics, Beijing Research Institute of Traumatology and Orthopaedics, Beijing Jishuitan Hospital, Beijing, China; ^2^ Department of Medical Oncology, National Cancer Center/National Clinical Research Center for Cancer/Cancer Hospital, Chinese Academy of Medical Sciences and Peking Union Medical College, Beijing, China; ^3^ Department of Orthopedics, Qilu Hospital of Shandong University, Jinan, China

**Keywords:** osteoarthritis, ferroptosis, TFRC, ATF3, CXCl2, JUN, chondrocyte, IL-1β

## Abstract

Osteoarthritis (OA), viewing as a degenerative aseptic inflammatory disease, is characterized by joint pain and inflammation that significantly affects the quality of patients’ life, especially for the elder. Although rapid progress has been achieved in elucidating the underlying mechanisms of OA occurrence and progression, there is still a lack of effective clinical therapeutics for OA patients. Currently the most common treatments including drug therapy and surgical operations are not very satisfactory in majority of cases, so it is worthy to explore new remedies. During the past few decades, a number of novel forms of regulated cell death have been reported widely, typified by ferroptosis, with its prominent features including reactive oxygen species (ROS) elevation, lipid peroxidation, iron accumulation and glutathione deprivation. Our study was designed to identify the functional roles of differentially expressed ferroptosis-related genes in OA, which were screened out by referring to GEO database *via* bioinformatics analyses. Human chondrocytes were applied to validate the above findings in the scenario of ferroptosis inhibitors administration. Results partially proved the consistency with bioinformatics analyses that ATF3 and TFRC were highly expressed in interleukin-1β (IL-1β)-stimulated chondrocytes whereas CXCL2 and JUN were downregulated. Besides, TFRC was firstly validated to be upregulated in IL-1β-stimulated chondrocytes, which could be reversed by ferroptosis inhibitors. In conclusion, our study reported two prominent ferroptosis-related genes, ATF3 and TFRC are upregulated in IL-1β-stimulated chondrocytes while CXCL2 and JUN are downregulated. And preliminary results demonstrated that TFRC might serve as an accomplice of ferroptosis process in IL-1β-stimulated chondrocytes and ferroptosis inhibitors have the potential to inhibit ROS in IL-1β-stimulated chondrocytes.

## Introduction

Osteoarthritis (OA) is a degenerative inflammatory disease with a clinical symptom of severe joint pain, which occurs mostly in the elder and is the most common type of arthritis. The etiology of OA still remains unclear, but many factors, such as increase of age, gender, genetic predisposition, obesity and joint misalignment seem to be associated with OA progress. Furthermore, OA is viewed to have complex pathophysiology affecting multiple joints and joint structures, as defined by the Osteoarthritis Research Society International (Katz et al., 2021). As reported by United States Centre for Disease Control and Prevention (CDC) that by 2040, an estimated 78 million adults in the United States will suffer confirmed arthritis, among whom two-thirds are women. Currently there is no ideal drug therapy for OA patients while non-steroidal anti-inflammatory drugs are still commonly used and surgery is also a reluctant choice when necessary. Interleukin-1β (IL-1β) is an inflammatory cytokine that is recognized to be highly differentially expressed in osteoarthritis compared to normal chondrocytes. The release of IL-1β promotes the production and release of several inflammatory mediators and catabolic factors, such as IL-6, cyclooxygenase-2 (COX-2), prostaglandin E2 (PGE2), tumor necrosis factor-α (TNF-α), and matrix metalloproteinases (MMPs), which contribute to chondrocytic dysfunction and extracellular matrix degradation (Shen et al., 2014). Inhibition of IL-1β and IL-1β-induced inflammatory response may be an effective strategy to treat OA. Therefore, it is urgent to explore some therapeutics to alleviate or even reverse OA progression.

**FIGURE 1:**
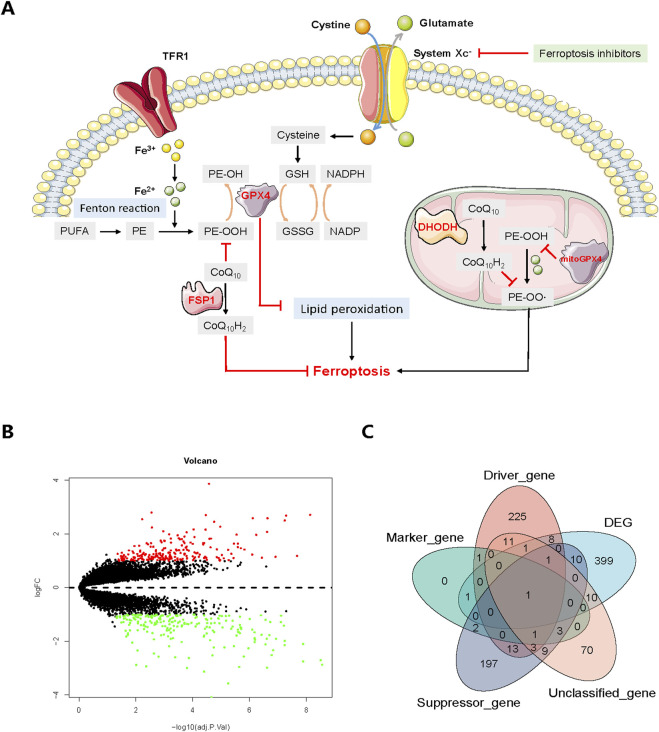
**(A)** Core mechanisms of ferroptosis. System Xc^−^-mediated cystine uptake, subsequent GSH production and GPX4 activation play a central role in protecting cells from ferroptosis. Alternatively, FSP1 inhibits ferroptosis by catalyzing the production of CoQ_10_H_2_ from CoQ_10_. Moreover, PUFA-phospholipids (PL-PUFAs) could be derived from PUFAs, which later produce PL-PUFA-OOH through Fenton reaction. TFR1 transports iron ion, which then participates the Fenton reaction in the form of ferrous ion. In the mitochondria, DHODH facilitates the generation of CoQ_10_H_2_ to suppress ferroptosis whereas the mitochondria GPX4 could restrain PE-OOH from producing PE-OO·, thus further arresting ferroptosis process. The red marks and black arrows indicate ferroptosis promotion and inhibition. **(B)** Volcano plot of DEGs between normal and osteoarthritis patients. Upregulated DEGs are indicated by red dots while downregulated DEGs are indicated by green. **(C)** Venn diagram showing the overlap between DEGs and ferroptosis-related genes. GSH, glutathione; GSSG, oxidized glutathione; NADPH, reduced nicotinamide adenine dinucleotide (phosphate); NADP, oxidized nicotinamide adenine dinucleotide (phosphate); PE, PL-PUFA.

During the past few decades, diverse types of regulated cell death (RCD) have been studied, including apoptosis, necroptosis, autophagy, ferroptosis (Dixon et al., 2012), pyroptosis (Wang et al., 2017) and recently reported cuproptosis (Tsvetkov et al., 2022). Ferroptosis, firstly identified in 2012 (Dixon et al., 2012), which functions in many physiological and disease events including inflammation (Sun et al., 2020), infection (Chen et al., 2021a) and cancerous diseases (Li et al., 2021a; Chen et al., 2021b; Qiu et al., 2022), is a novel form of RCD morphologically, biochemically and genetically distinct from apoptosis, necroptosis and autophagy (Dixon et al., 2012). The characteristics of ferroptosis comprise reactive oxygen species (ROS) elevation, lipid peroxidation, iron accumulation and glutathione (GSH) deprivation (Dixon et al., 2012). Over the years, great breakthroughs have been accomplished in uncovering the underlying mechanisms of ferroptosis. Generally speaking, classical pathways of ferroptosis include system Xc^−^/GSH/glutathione peroxidase 4 (GPX4) protection pathway, P53 pathway (Jiang et al., 2015), ferroptosis suppressor protein 1 (FSP1)/coenzyme Q_10_ (CoQ_10_) protection pathway (Bersuker et al., 2019), iron metabolism pathway through Fenton reaction, dihydroorotate dehydrogenase (DHODH)-mediated protection pathway in mitochondria (Mao et al., 2021) and polyunsaturated fatty acids (PUFAs)-governed lipid metabolism pathway; in the meanwhile glutamine metabolic pathway also plays a vital role in regulating ferroptosis (Li et al., 2020). The system Xc^−^, composed of solute carrier family 7 member 11 (SLC7A11) and solute carrier family 3 member 2 (SLC3A2), resides on the cell membrane helping to transfer cystine as well as glutamate. Specifically in cytoplasm, GPX4 could inhibit lipid peroxidation together with GSH, while on the other hand, FSP1 catalyzes CoQ_10_ to generate CoQ_10_H_2_, also known as ubiquinol to combat ferroptosis. Moreover, PUFA-phospholipids (PL-PUFAs) could be derived from PUFAs, which later produce PL-PUFA-OOH through Fenton reaction. During this process, transferrin receptor 1 (TFR1) encoded by transferrin receptor (TFRC) gene could transport iron ion, which then becomes ferrous ion to participate in Fenton reaction. Of note, in the mitochondria DHODH facilitates the generation of CoQ_10_H_2_ to suppress ferroptosis whereas the mitochondria GPX4 could restrain PE-OOH from producing PE-OO·, thus further arresting ferroptosis process (Figure 1A). Ferroptosis inducers, including but not be limited to erastin and RSL3, have been widely tested in cancer treatment (Yang et al., 2014; Li et al., 2020; Ghoochani et al., 2021), showing promising antitumor efficacy. Additionally, ferroptosis inhibitors, like ferrostatin-1 (Fer-1), vitamin E (Vit E), deferasirox (DFX) and liproxststatin-1, could alleviate inflammation-related features involved in acute lung injury (Liu et al., 2020), neuron degeneration (Chen et al., 2015), neuroinflammation (Cao et al., 2021), and lipid peroxidation (Jomen et al., 2022). Therefore, considering the crucial role of inflammation in both OA and ferroptosis, this study was designed to examine the effects of ferroptosis and its inhibitors on IL-1β-stimulated osteoarthritis, aiming to provide some valuable clues for future explorations.

## Materials and methods

### Data acquisition and processing

The Gene Expression Ominibus (GEO) database (https://www.ncbi.nlm.nih.gov/geo/) was queried. Two microarray datasets GSE55235 and GSE55457 from the same study were analyzed which were detected under the same platform (GPL96, HG-U133A). A total of 20 normal synovial tissues from healthy controls (HC) and 20 samples of synovium from OA patients (OA) were enrolled, with 10 from each dataset, respectively. The two probe expression matrix files were downloaded and the data were log2 transformed using the R software (version 4.2.0). Then the probes were annotated using the Perl software (version 5.32.1) with the platform annotation file and then well-annotated probes were retained to get gene expression files. Multiple probes corresponding to one gene were aggregated by the average expression value, then two gene expression files were merged using the Perl software and were normalized using the sva package in R.

### Differentially expressed gene and ferroptosis-related gene analyses

Differentially expressed genes (DEGs) between HC and OA samples were screened out by the limma package (Ritchie et al., 2015). Statistically significant DEGs were defined with criteria of an adjust *P* (adj. *P*) < 0.05 and absolute log2-fold change (|log2-FC|) > 1. The DEGs were presented by a volcano plot and the pheatmap package was utilized to plot the heatmap of DEGs between different patients from different datasets. Ferroptosis-related genes (FRGs), including driver genes, suppressor genes, marker genes and unclassified genes, were harvested from the FerrDb website (http://www.zhounan.org/ferrdb/current/). And the overlap genes between DEGs and FRGs, namely, the differentially expressed ferroptosis-related genes (DFGs) were identified and screened out by taking the intersection of the DEG list and the FRG list while a venn plot was drawn as well using the venn package and the VennDiagram package.

### Functional enrichment analysis and protein-protein interaction network construction

The clusterProfiler package (Wu et al., 2021) was deployed to conduct Gene Ontology (GO) and Kyoto Encyclopedia of Genes and Genomes (KEGG) pathway analyses for both DEGs and DFGs. Annotation of cellular components (CCs), biological processes (BPs) and molecular functions (MFs) were determined using the GO enrichment analysis under the condition of *p* < 0.05 and adj. *p* < 0.05. KEGG analysis of both DEGs and DFGs was carried out with *p* < 0.05 and adj. *p* < 1. To explore the interaction of proteins encoded by DEGs and DFGs, as well as to identify hub genes, protein-protein interaction (PPI) networks of DEGs and DFGs were constructed using the online Search Tool for the Retrieval of Interacting Genes/Proteins (STRING) (https://cn.string-db.org/) tool. DEGs and DFGs were imported and the minimum required interaction score was set at a high confidence of 0.7 and false discovery rate (FDR) stringency at 5% while the disconnected nodes were hidden from the PPI network. Then the top 30 DEGs and all DFGs were analyzed for nodes and edges using R software.

### Reagents and cell culture

Ferroptosis inhibitors including Ferrostatin-1 (T6500; CAS: 347174-05-4), α-Vitamin E (T1648; CAS: 59-02-9) and Deferasirox (T1457; CAS: 201530-41-8) were purchased from TargetMol, China. Fer-1 and DFX were dissolved in dimethyl sulfoxide (DMSO) solution at the concentration of 10 mM while Vit E was dissolved in DMSO at the concentration of 100 mM. IL-1β was obtained from Sigma, Uited States (SRP3083). Human primary chondrocytes together with the culture system were bought from the Beijing Beina Chuanglian Institute of Biotechnology. Cells were cultured in Dulbecco’s modified Eagle’s medium (DMEM)/F-12 supplemented with 10% fetal bovine serum and 1% penicillin-streptomycin in a 37°C incubator under condition of 5% CO_2_. After reaching 80%-90% confluence, cells were digested with trypsin (Gibco, United States) and passaged twice per week at a split ratio of 1:3. Cells at passages 3-4 were used for the subsequent experiments.

### Quantitative real-time PCR (qRT-PCR) analysis

Chondrocytes were seeded into six-well plates and incubated in complete culture medium. MiniBEST Universal RNA Extraction Kit (TaKaRa, China) was used to extract total RNA from chondrocytes in accordance with the manufacturer’s instructions. PrimeScript™ RT reagent Kit with gDNA Eraser (TaKaRa, China) was used to synthesize cDNA and to eliminate DNA contamination. Real-time PCR was carried out using the Applied Biosystems 7,500 Real Time PCR System and TB Green Premix Ex Taq (TaKaRa, China) according to the manufacturer’s instructions. GAPDH was used as internal reference. The data were analyzed to calculate the relative gene expression by the comparison Ct (2^−ΔΔCT^) method. The sequences of the gene primers are listed in Table 1.

**TABLE 1:** The primers used for quantitative real-time PCR analyses.

Target	Sequence (5′-3′)
JUN-F	TCC​AAG​TGC​CGA​AAA​AGG​AAG
JUN-R	CGA​GTT​CTG​AGC​TTT​CAA​GGT
TFRC-F	GGC​TAC​TTG​GGC​TAT​TGT​AAA​GG
TFRC-R	CAG​TTT​CTC​CGA​CAA​CTT​TCT​CT
ATF3-F	CTG​GAA​AGT​GTG​AAT​GCT​GAA​C
ATF3-R	ATT​CTG​AGC​CCG​GAC​AAT​AC
CXCL2-F	ACG​GCA​GGG​AAA​TGT​ATG​TGT
CXCL2-R	CTG​CTC​TAA​CAC​AGA​GGG​AAA​C
SLC7A11-F	TCA​TTG​GAG​CAG​GAA​TCT​TCA
SLC7A11-R	TTC​AGC​ATA​AGA​CAA​AGC​TCC​A
NRF2-F	TCC​AGT​CAG​AAA​CCA​GTG​GAT
NRF2-R	GAA​TGT​CTG​CGC​CAA​AAG​CTG
GAPDH-F	AAG​GGT​CAT​CAT​CTC​TGC​CC
GAPDH-R	GTG​AGT​GCA​TGG​ACT​GTG​GT

### Cell viability assay

Cell Counting Kit-8 (CCK8) (Dojindo Molecular Technology) was used to detect cell viability. Human primary chondrocytes were seeded into 96-well plate at a density of 5,000 cells/well. After 12 h, fresh medium containing 10 ng/mL IL-1β with 1 μM Fer-1, 1 μM DFX and 100 μM Vit E was added to each well according to previous reports (Bhatti et al., 2013). Cells were incubated for 12 h, 24 h and 48 h in a humidified incubator with 5% CO_2_ at 37°C. CCK8 kit was used to evaluate the cytotoxicity of Fer-1, DFX and Vit E. CCK8 reagent was added into each well and the cells were cultured at 37°C for 2 h. The absorbance of each well at 450 nm was measured by a microplate reader. Cell viability of the control group was set at 100%.

### ROS detection assay

Chondrocytes were firstly seeded into 6-well plates at a density of 3^*^10^5^ cells/well. After 24 h, chondrocytes were treated with 1 μM ferrostain-1, 1 μM deferasirox and 100 μM α-Vitamin E in the presence 10 ng/mL IL-1β for 48 h (Bhatti et al., 2013). The intracellular ROS and lipid-ROS levels were measured with the DCFH-DA fluorescent probe (Beyotime, China) where the Rosup was used as positive control complying with the manufacturer’s instructions. In brief, chondrocytes were washed with PBS three times and treated with 10 μM DCFH-DA for 20 min at 37°C in the dark. After incubation, cells were washed with PBS and observed under the fluorescence microscope.

### Statistical analysis

The data are presented as the mean ± standard deviation (SD) as indicated. For statistical analysis, the differences among groups were calculated by one-way ANOVA after testing for the homogeneity of variance and data from the same group were evaluated by Student’s t-test. All experiments were repeated at least three times. A value of *p* < 0.05 was considered statistically significant.

## Results

### Identification of DEGs and DFGs

**FIGURE 2:**
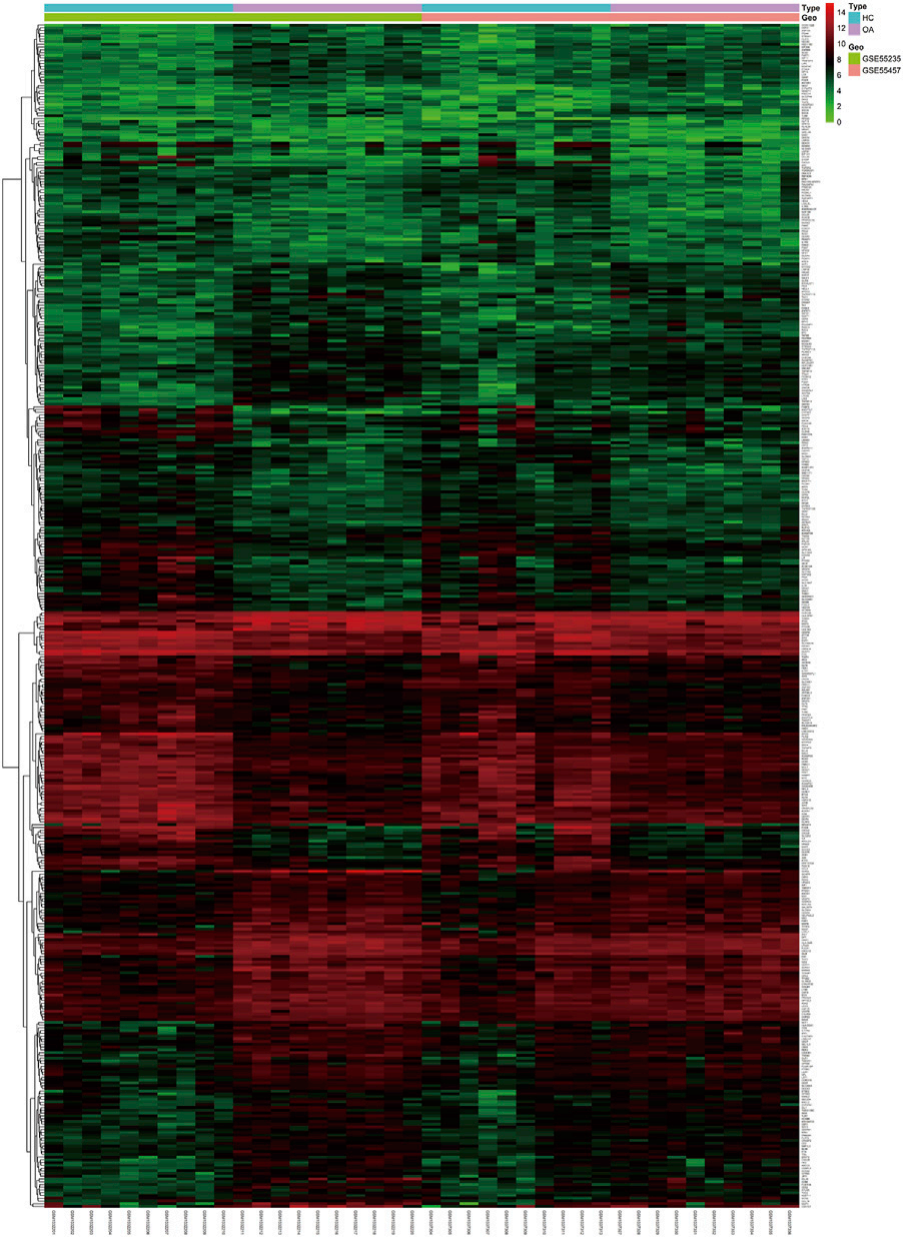
Heatmap of DEGs from two GEO datasets between healthy control and osteoarthritis patients. HC: healthy control; OA: osteoarthritis.

After compiling the integrated dataset, a total of 431 DEGs were finally identified between OA and HC samples (Figure 1B), including 214 downregulated DEGs and 217 upregulated DEGs. The downregulated DEGs included vascular endothelial growth factor A (VEGFA) (log2-FC = −2.33; adj. *p* < 0.001), activating transcription factor 3 (ATF3) (log2-FC = −2.52; adj. *p* < 0.001), Jun proto-oncogene (JUN) (log2-FC = −2.99; adj. *p* < 0.001), C-X-C motif chemokine ligand 2 (CXCL2) (log2-FC = −2.96; adj. *p* < 0.001), early growth response 1 (EGR1) (log2-FC = −1.01; adj. *p* < 0.001) and TFRC (log2-FC = −1.16; adj. *p* = 0.002). The upregulated DEGs encompassed C-X3-C motif chemokine receptor 1 (CX3CR1) (log2-FC = 2.71; adj. *p* < 0.001), C-X-C motif chemokine ligand 12 (CXCL12) (log2-FC = 1.47; adj. *p* < 0.001), neural EGFL like 1 (NELL1) (log2-FC = 2.17; adj. *p* < 0.001), matrix metallopeptidase 1 (MMP1) (log2-FC = 2.80; adj. *p* = 0.003) and matrix metallopeptidase 9 (MMP9) (log2-FC = 1.39; adj. *p* = 0.017) (Supplementary File S1). The heatmap showed the DEG expression profile of 431 DEGs from 20 HC and 20 OA samples (Figure 2) while the venn plot determined 32 DFGs that were related to ferroptosis, in particular for TFRC as the potential pivotal hub gene involved in all 4 groups and JUN as a suppressor gene (Figure 1C) (Supplementary File S2).

### GO and KEGG enrichment analyses

**FIGURE 3:**
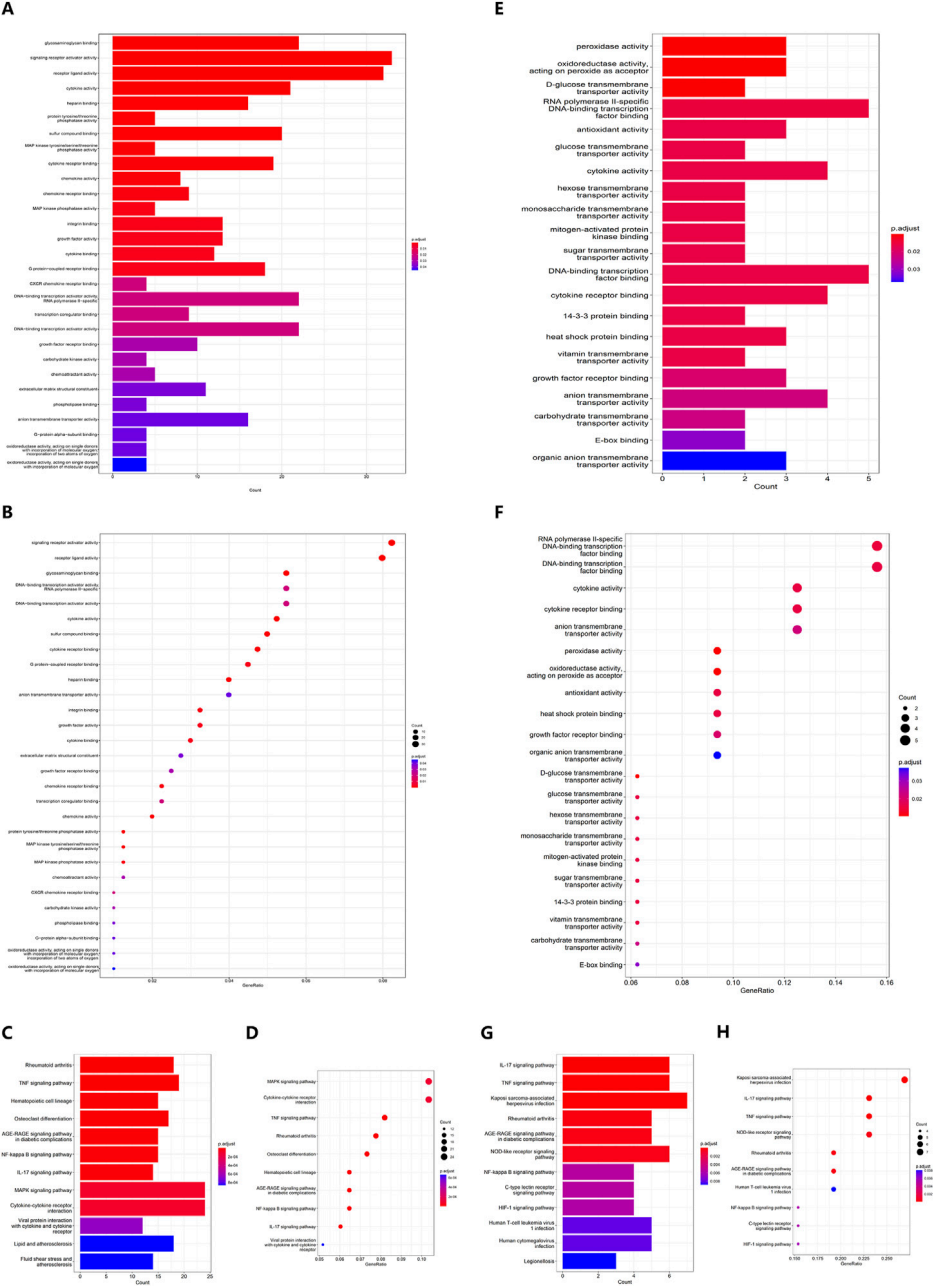
**(A)** Barplot and **(B)** Bubble plot of DEG GO enrichment analysis. **(C)** Barplot and **(D)** Bubble plot of DEG KEGG pathway enrichment analysis. **(E)** Barplot and **(F)** Bubble plot of DFG GO enrichment analysis. **(G)** Barplot and **(H)** Bubble plot of DFG KEGG pathway enrichment analysis.

GO analysis showed that 33 DEGs were enriched upon signaling receptor activator activity (adj. *p* < 0.001), which ranked first among all CCs, BPs and MFs. And receptor ligand activity is the second most enriched compartment with 32 DEGs (adj. *p* < 0.001). Other biology events, including glycosaminoglycan binding and cytokine activity, were also enriched with at least 20 DEGs, indicating a delicate interaction network between different cell types and cytokines in OA patients (Figures 3A,B) (Supplementary File S3). KEGG analysis of DEGs confirmed that both MAPK signaling pathway (adj. *p* < 0.001) and cytokine-cytokine receptor interaction (adj. *p* < 0.001) were enriched with 24 DEGs, followed by TNF signaling pathway with 19 DEGs (adj. *p* < 0.001), which was consistent with the inflammatory microenvironment in OA patients (Figures 3C,D) (Supplementary File S4).

As for the DFGs, RNA polymerase II-specific DNA-binding transcription factor binding together with DNA-binding transcription factor binding are the most enriched compartments in GO analysis, both with 5 out of 32 DFGs (Figures 3E,F) (Supplementary File S5) while surprisingly Kaposi sarcoma-associated herpesvirus infection was the top pathway enriched with 7 DFGs (adj. *p* < 0.001), prevailing over IL-17 signaling pathway, TNF signaling pathway and NOD-like receptor signaling pathway, all of which were enriched with 6 DFGs (Figures 3G,H) (Supplementary File S6).

### PPI network analysis

**FIGURE 4:**
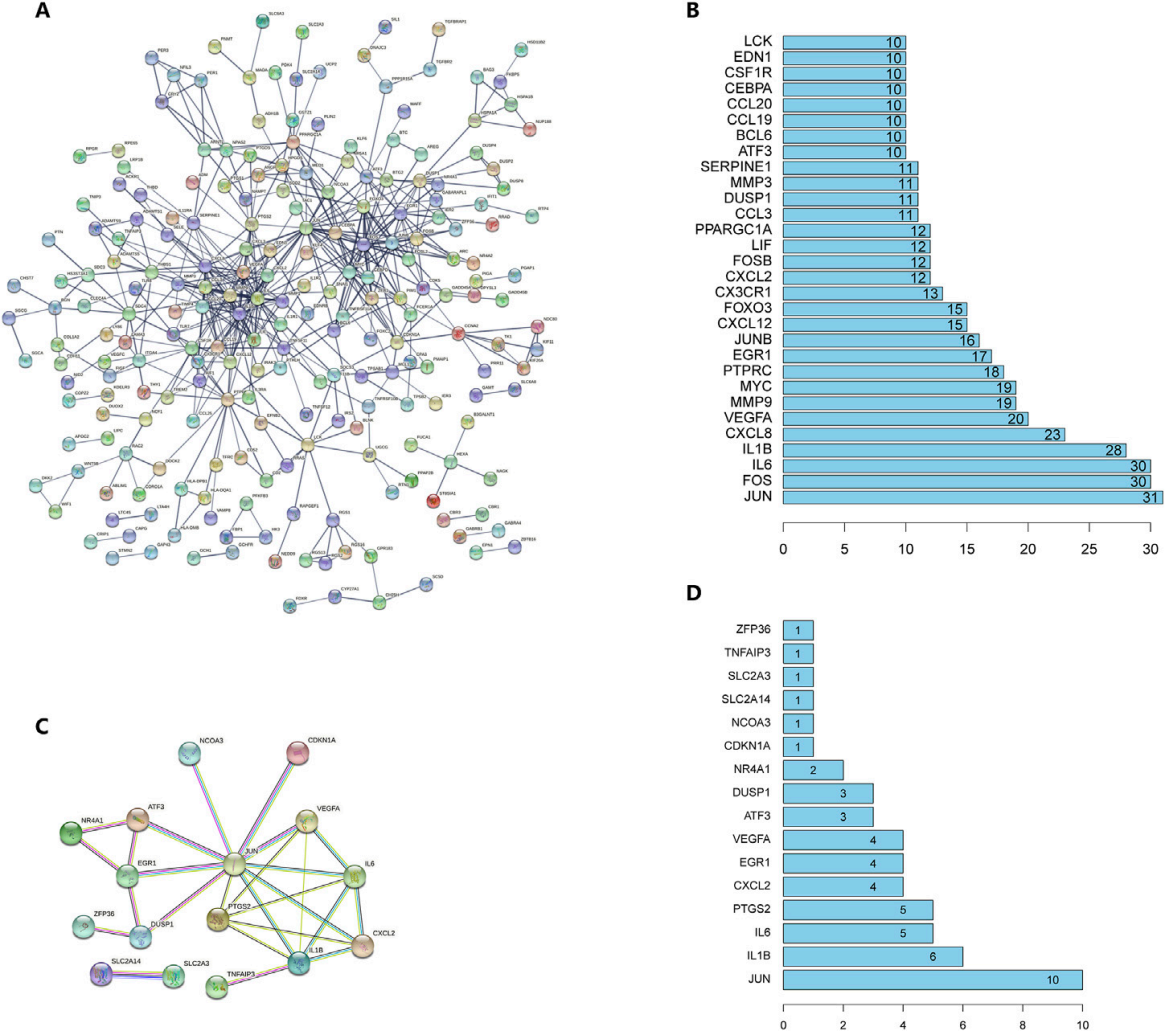
**(A)** PPI network of DEGs. A node represents a protein and an edge represents an interaction between two proteins. For node content, empty nodes represent proteins of unknown 3D structure and filled nodes represent proteins with known or predicted 3D structure. **(B)** The number of interactions of top 30 proteins with each other. **(C)** PPI network of DFGs. Different edge colors represent different types of associations. **(D)** The number of interactions of all differentially-expressed ferroptosis-related proteins with each other.

PPI network visualization was harvested from STRING database and the tabular file showing the edges between different protein nodes was acquired as well. In DEG-coding-protein interaction network, after removing proteins that were not connected to others, 511 edges representing 511 pairs of protein-protein-interaction between 226 nodes representing 226 proteins were displayed in the interaction diagram (Figure 4A) (Supplementary File S7). The barplot exhibited the top 30 genes that harbored the most interaction relations, where c-JUN encoded by JUN gene could interact with 31 other proteins and many inflammatory cytokines could interact with each other as expected (Figure 4B); for example, IL1B-encoding-cytokine IL-1β and IL6-encoding-cytokine IL-6 could interact with more than 20 proteins including each other. Furthermore, after hiding the disconnected nodes, a total of 16 DFGs were discovered to possess 26 interaction pairs (Supplementary File S8) and c-JUN was demonstrated to be associated with 10 proteins including IL-1β, IL-6, CXCL2 and ATF3, *etc.* (Figures 4C,D). Thereafter, it seems that JUN gene can serve as an important hub gene in OA patients as compared to healthy donor.

### Expression of several key DEGs between OA and normal chondrocytes

**FIGURE 5:**
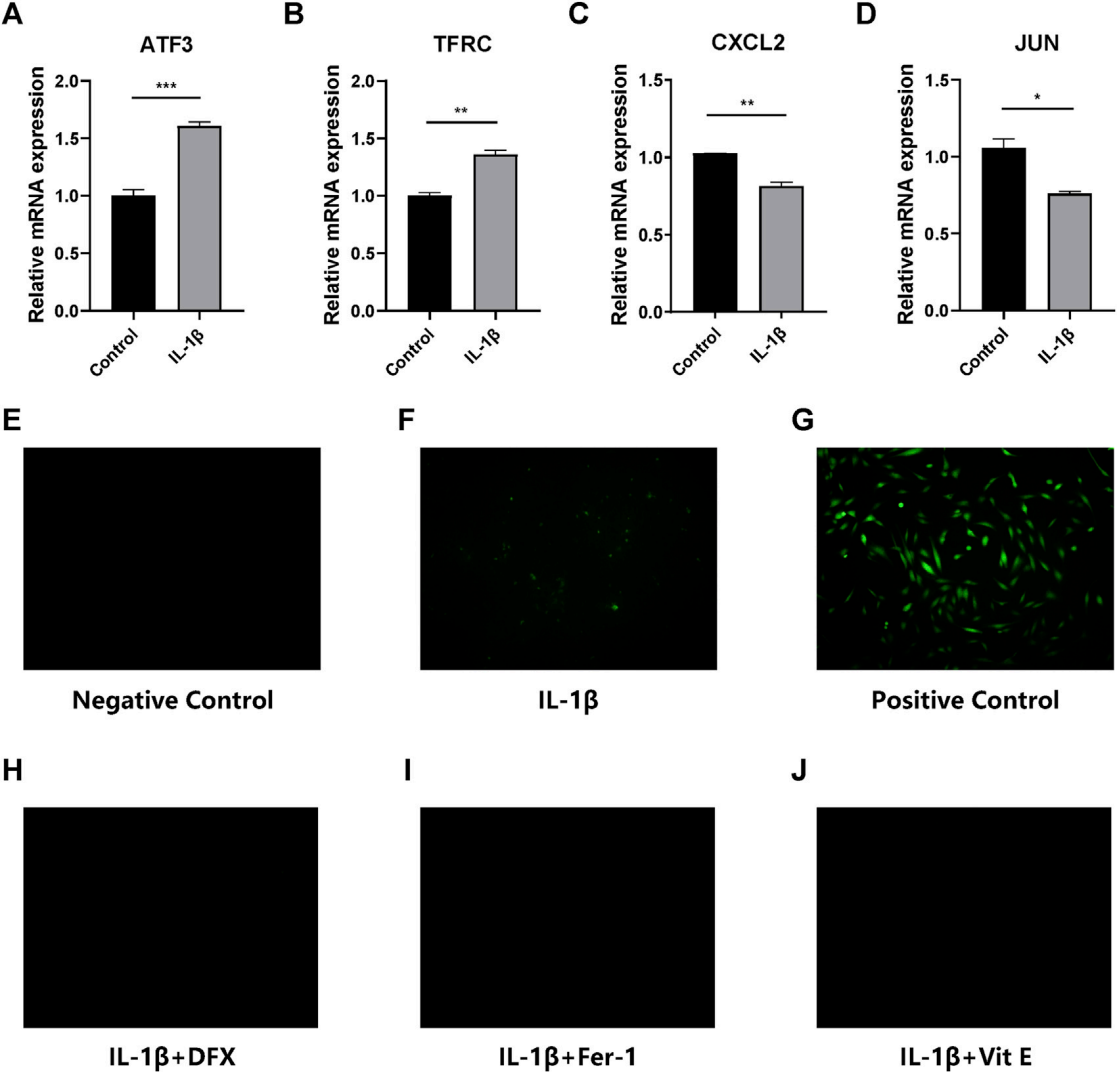
**(A–D)** Quantitative real-time PCR analyses of ATF3, TFRC, CXCL2 and JUN. ^*^ *p* < 0.05; ^**^ *p* < 0.01; ^***^ *p* < 0.001. **(E–J)** ROS detection of the control groups and cells receiving IL-1β alone, IL-1β + deferasirox, IL-1β + ferrostatin-1 and IL-1β + vitamin E. The green fluorescence represents ROS detected in chondrocytes. DFX: deferasirox; Fer-1: ferrostatin-1; Vit E: vitamin E.

Chondrocytes stimulated with IL-1β for 48 h were harvested and RNA were extracted to analyze DEGs, with untreated chondrocytes serving as control. Notably, ATF3, CXCL2, TFRC and JUN were all significantly differentially expressed after IL-1β administration in chondrocytes. Specifically, the expression of ATF3 induced by IL-1β was more than 1.5 times higher than that in normal chondrocytes (*p* < 0.001) (Figure 5A). Similarly, the expression of TFRC was slightly increased by IL-1β compared with normal chondrocytes (*p* = 0.008) (Figure 5B). On the contrary, CXCL2 and JUN were both downregulated obviously in IL-1β-stimulated chondrocytes, with *p* values of 0.006 for CXCL2 (Figure 5C) and 0.019 for JUN (Figure 5D), respectively.

### Ferroptosis is suppressed in IL-1β-stimulated chondrocytes by ferroptosis inhibitors

**FIGURE 6:**
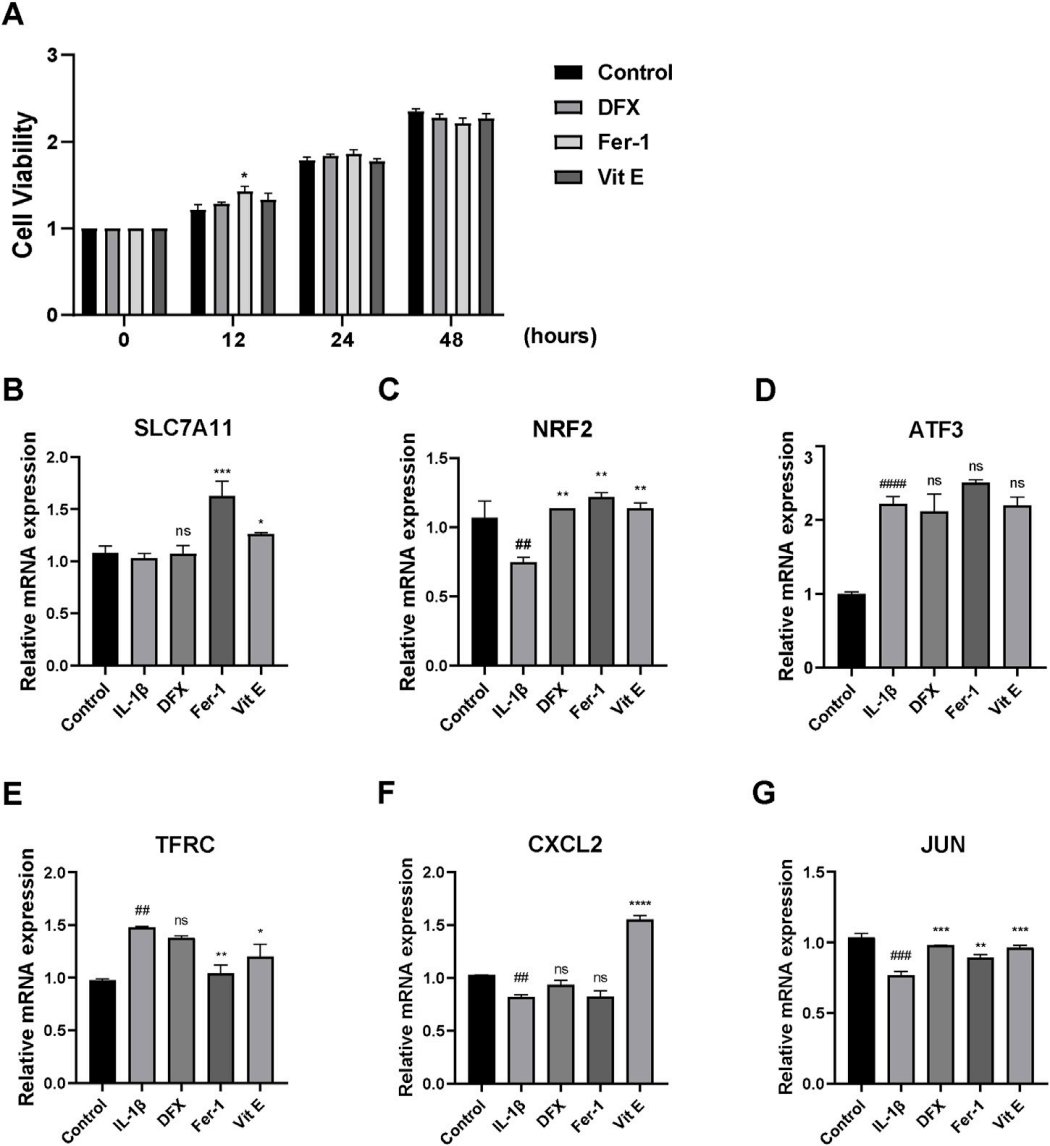
**(A)** CCK8 analysis results of cell viability of chondrocytes receiving IL-1β alone, IL-1β + deferasirox, IL-1β + ferrostatin-1 and IL-1β + vitamin **(E)**. **(B–C)** Quantitative real-time PCR analyses of SLC7A11 and NRF2. **(D–G)** Quantitative real-time PCR analyses of ATF3, TFRC, CXCL2 and JUN in the control group and chondrocytes receiving IL-1β alone, IL-1β + deferasirox, IL-1β + ferrostatin-1 and IL-1β + vitamin E. DFX: deferasirox; Fer-1: ferrostatin-1; Vit E: vitamin E; ns: not significant compared to IL-1β treatment group; ^##^ significant compared to the control group, *p* < 0.01; ^###^ significant compared to the control group, *p* < 0.001; ^####^ significant compared to the control group, *p* < 0.0001; ^*^ *p* < 0.05 compared to IL-1β treatment group; ^**^ *p* < 0.01 compared to IL-1β treatment group; ^***^ *p* < 0.001 compared to IL-1β treatment group; ^****^ *p* < 0.0001 compared to IL-1β treatment group.

ROS detection, CCK8 and qRT-PCR were performed to determine whether Fer-1, Vit E and DFX could inhibit ferroptosis in IL-1β-stimulated chondrocytes. The fluorescent dye was used to represent ROS level in chondrocytes. IL-1β increased ROS level in chondrocytes compared with normal chondrocytes, but its ability to induce ROS was not as potent as strong oxidant, namely, the positive control (Figures 5E–G). Nevertheless, three ferroptosis inhibitors could significantly protect IL-1β-stimulated chondrocytes from ferroptosis, as well as decrease the ROS level in IL-1β-stimulated chondrocytes (Figures 5H–J). According to the CCK8 results, cell viability in all treatment groups was comparable, suggesting a tolerable toxicity of the ferroptosis inhibitors while ferrostatin-1 treatment showed significantly elevated cell viability at 12 h (*p* = 0.022) (Figure 6A). Two FRGs, SLC7A11 and NFE2 like bZIP transcription factor 2 (NRF2) were analyzed. It could be observed that ferrostatin-1 and vitamin E significantly upregulated the expression of SLC7A11 (*p* < 0.001; *p* = 0.045, respectively), the gatekeeper of classical ferroptosis protection pathway (Figure 6B). However, deferasirox seemed not to impact SLC7A11 expression. The expression of endogenous antioxidant NRF2 was significantly upregulated by all these three ferroptosis inhibitors (*p* = 0.004 for DFX; *p* = 0.002 for Fer-1; *p* = 0.004 for Vit E, respectively) while IL-1β could significantly downregulate NRF2 expression (*p* = 0.009) (Figure 6C), implying that ferroptosis might play an important role in the onset and development of OA.

### Roles of key DFGs in ferroptosis of IL-1β-stimulated chondrocytes

Four key DFGs, ATF3, TFRC, CXCL2 and JUN were then further analyzed. However no significant discrepancies were observed in ATF3 expression between different ferroptosis inhibitor-treated groups compared with IL-1β treatment (Figure 6D). Incredibly, TFRC was more expressed in IL-1β-stimulated chondrocytes (*p* = 0.002), and ferrostatin-1 as well as vitamin E could significantly decrease TFRC expression in IL-1β-stimulated chondrocytes (*p* = 0.003; *p* = 0.023, respectively). Deferasirox again showed no effect (Figure 6E). As for the results of CXCL2, only vitamin E could significantly upregulate its expression (*p* < 0.001) (Figure 6F). Besides, all three ferroptosis inhibitors increased JUN expression (*p* < 0.001 for DFX; *p* = 0.005 for Fer-1; *p* < 0.001 for Vit E) whereas IL-1β significantly lowered the expression of JUN compared to normal chondrocytes (*p* < 0.001) (Figure 6G).

## Discussion

As a common disease of joints, the incidence of OA has increased in recent years. Previous studies have demonstrated that various types of chondrocyte death, inflammation and oxidative stress contribute to the progression of OA (Loeser et al., 2016; Feng et al., 2019; Sanchez-Lopez et al., 2022). Meanwhile as a chronic degenerative disease, OA affects the life quality of the aged in daily, placing a heavy burden onto both patients and the society, but there is still a lack of effective method in treatment. Ferroptosis, a novel form of regulated cell death, is featured by the iron-dependent accumulation of lethal lipid ROS at overwhelming levels (Yang and Stockwell, 2016), partially resembling one of the characteristics of osteoarthritis. Previous studies have proved that IL-1β had positive effects on chondrocyte ferroptosis, such as its ability to inhibit SLC7A11 and GPX4, induce the excessive expression of P53 and acyl-CoA synthetase long-chain family member 4 (ACSL4), cause ROS to accumulate, and increase malondialdehyde production in chondrocytes. IL-1β and ferric ammonium citrate (FAC) induced ferroptosis related protein expression changes in chondrocytes (Yao et al., 2021; Guo et al., 2022). Therefore, in our study, differentially expressed genes between normal and IL-1β-stimulated chondrocytes were analyzed and four prominent ferroptosis-related genes, ATF3, TFRC, CXCL2 and JUN, were identified to be differentially expressed. According to the experimental results, CXCL2 and JUN were downregulated in IL-1β-stimulated chondrocytes while conversely, ATF3 and TFRC were upregulated, which is opposite from the results of bioinformatics analysis, probably due to the fact that osteoarthritis patients from GSE55235 and GSE55457 datasets received non-steroidal anti-inflammatory drugs or the sequencing deviations.

Specifically, although both ATF3 and TFRC were downregulated in OA samples from bioinformatics analyses, many other researches have found that ATF3 is indispensable for the detrimental effects of IL-1β (Rhee et al., 2017; Li et al., 2021b), which was indicated to participate in pathogenesis of OA through modulating inflammatory cytokine expression in chondrocytes (Iezaki et al., 2016) and could serve as a potential diagnostic marker of early-stage OA based on another bioinformatics analysis result (Yang et al., 2022). And to our knowledge, the relationship between OA and TFRC was not reported before; so we firstly identified that TFRC was upregulated in IL-1β-stimulating chondrocytes, simulating the pathophysiological state of patients with osteoarthritis. Moreover, as a well-known proinflammatory cytokine secreted by mast cells and macrophages (De Filippo et al., 2013), CXCL2 was unexpectedly downregulated in IL-1β-stimulating chondrocytes, which was consistent with another previously reported article (Cai et al., 2020), and the same is true of JUN expression (Cai et al., 2020).

Additionally in our study, results showed that all the three ferroptosis inhibitors could reduce ROS accumulation in chondrocytes, meanwhile impacting on the expression levels of some key ferroptosis-related genes. Fer-1 and Vit E increased cellular uptake of cysteine by up-regulating the expression of SLC7A11, the component of system Xc^−^, resulting in an increase in glutathione peroxidase activity and cell antioxidant capacity, thereby inhibiting ferroptosis of chondrocytes. As a key transcription factor of antioxidant response, NRF2 has been considered as an important therapeutic target for oxidative stress-related cancers (Sun et al., 2016; Li et al., 2021a). In the present study, all the three ferroptosis inhibitors upregulated NRF2 to drive antioxidant gene expression. But other from apoptosis, the golden standard has not been established to evaluate ferroptosis. However, according to the definition of ferroptosis from the Nomenclature Committee on Cell Death, we validated the induction of chondrocyte ferroptosis by IL-1β, and preliminary evidence has shown that DFX, Fer-1 and Vit E might inhibit ferroptosis and delay OA progression *in vitro*.

Intriguingly, TFRC was significantly downregulated in IL-1β-stimulated chondrocytes receiving ferroptosis inhibitors including Fer-1 and Vit E, suggesting an accomplice role of TFRC in ferroptosis under osteoarthritic condition which was in line with other studies. In most cases, especially in cancerous diseases, it was reported that elevation of TFRC could sensitize cancer cells to ferroptosis (Lu et al., 2021; Zhu et al., 2021), as well as other cell types (Zhuang et al., 2021; Wei et al., 2022). Yet in another article studying the effect of transferrin receptor 1 (TFR1) on skeletal muscle regeneration, which was encoded by TFRC, it seemed that TFR1 ablation activated ferroptosis, thus arresting skeletal muscle regeneration process (Ding et al., 2021). And here in the present study, we preliminarily demonstrated that TFRC was upregulated in IL-1β-stimulated chondrocytes and that TFRC might play a role in promoting ferroptosis in IL-1β-stimulated chondrocytes, which could serve as a potential target for OA treatment.

## Conclusion

In summary, our research has indicated that chondrocyte ferroptosis is a significant factor that might promote the onset and development of OA, and DFX, Fer-1 and Vit E could protect chondrocytes from ferroptosis and delay the progression of OA by inhibiting chondrocyte ferroptosis. We also firstly identified that TFRC was upregulated in osteoarthritis, and could sensitize cells to ferroptosis, which might serve as a potential target for OA treatment in the future.

## Data Availability

The original contributions presented in the study are included in the article/Supplementary Material, further inquiries can be directed to the corresponding author.
